# Interventions to improve adherence to cardiovascular disease guidelines: a systematic review

**DOI:** 10.1186/s12875-015-0341-7

**Published:** 2015-10-22

**Authors:** Rebecca A. Jeffery, Matthew J. To, Gabrielle Hayduk-Costa, Adam Cameron, Cameron Taylor, Colin Van Zoost, Jill A. Hayden

**Affiliations:** Faculty of Medicine, Dalhousie University, Mailbox 354, 5849 University Avenue, Halifax, NS Canada B3H 4R2; Department of Science, St. Mary’s University, Halifax, Canada; Department of Medicine, Dalhousie University, Halifax, Canada; Department of Community Health and Epidemiology, Dalhousie University, Halifax, Canada

**Keywords:** Clinical practice guidelines, Cardiovascular disease, Adherence, Systematic review

## Abstract

**Background:**

Successful management of cardiovascular disease (CVD) is impaired by poor adherence to clinical practice guidelines. The objective of our review was to synthesize evidence about the effectiveness of interventions that target healthcare providers to improve adherence to CVD guidelines and patient outcomes.

**Methods:**

We searched PubMed, EMBASE, Cochrane Library, PsycINFO, Web of Science and CINAHL databases from inception to June 2014, using search terms related to adherence and clinical practice guidelines. Studies were limited to randomized controlled trials testing an intervention to improve adherence to guidelines that measured both a patient and adherence outcome. Descriptive summary tables were created from data extractions. Meta-analyses were conducted on clinically homogeneous comparisons, and sensitivity analyses and subgroup analyses were carried out where possible. GRADE summary of findings tables were created for each comparison and outcome.

**Results and Discussion:**

We included 38 RCTs in our review. Interventions included guideline dissemination, education, audit and feedback, and academic detailing. Meta-analyses were conducted for several outcomes by intervention type. Many comparisons favoured the intervention, though only the adherence outcome for the education intervention showed statistically significant improvement compared to usual care (standardized mean difference = 0.58 [95 % confidence interval 0.35 to 0.8]).

**Conclusions:**

Many interventions show promise to improve practitioner adherence to CVD guidelines. The quality of evidence and number of trials limited our ability to draw conclusions.

**Electronic supplementary material:**

The online version of this article (doi:10.1186/s12875-015-0341-7) contains supplementary material, which is available to authorized users.

## Background

Cardiovascular disease (CVD) is a leading cause of death in Canada [1]. Successful management of CVD involves not only the treatment of a specific disease, but also treating and preventing risk factors for CVD, including diabetes, dyslipidemia and hypertension [1–3]. However, the management of CVD is complicated by the large number of clinical practice guidelines available for conditions that contribute to this disease. An article by Ray et al. noted there are also discrepancies in recommendations across guidelines, potentially contributing to low adherence rates [2, 4, 5]. A harmonized guideline by Tobe et al*.* (2011) found there are over 400 recommendations for managing risk factors for heart disease [3].

Given the complexity of the management of this illness, it is imperative that practitioners use guidelines, and the most appropriate guidelines, in caring for patients with CVD and risk factors for CVD. The impact of guideline implementation has been illustrated previously; a review by Grimshaw and Russell found that using guidelines improved clinical practice [6]. Despite evidence to support the use of guidelines, there remains a gap in their implementation [7].

The dissemination of guidelines alone has little to no effect on practice [8], thus many studies have investigated interventions of varying intensity to increase the uptake of clinical practice guidelines. Numerous studies of interventions to improve the uptake of guidelines in CVD prevention are available. However, their overall impact on guideline adherence and clinical outcomes is unclear. Unverzagt et al. [9] published a systematic review on a similar topic that focused on primary care physicians’ adherence to guidelines, wherein they demonstrated these interventions can have an impact on adherence outcomes. It is important to determine the effect of these interventions on other healthcare providers, as well as determine the impact of these interventions on clinical outcomes, which is yet to be addressed in the literature to our knowledge.

We identified and synthesized the available research evidence about the effectiveness of interventions that target healthcare providers to improve adherence to CVD prevention and treatment guidelines and clinical outcomes. Our secondary objective was to explore characteristics of guideline implementation interventions and contexts that are associated with increased effectiveness. This leads to our research question: what is the most effective intervention to improve the implementation of, uptake of, or adherence to cardiovascular disease-related clinical practice guidelines by healthcare providers in randomized controlled trials?

## Methods

As this research did not involve the collection of primary data, we did not seek ethics approval. This review has been registered with PROSPERO 2014:CRD42014010111. Available from http://www.crd.york.ac.uk/PROSPERO/display_record.asp?ID=CRD42014010111

### Search

A systematic search was conducted using search terms related to “adherence” and “clinical practice guidelines”, which was refined with the help of a medical librarian. We searched the following databases: PubMed, EMBASE, Cochrane Library (including CENTRAL, DARE and HTAs), PsycINFO, Web of Science and CINAHL (all available years, up to June 2014). Grey literature was also searched, including clinicaltrials.gov to identify potential new studies, ICTRP registry database, and ProQuest thesis database. Our search strategy did not impose any limits on language of publication (Additional file 1).

### Inclusion criteria

#### Study design

The included studies were limited to randomized controlled trials (RCTs). We included all types of RCTs, including cluster RCTs, and nested designs.

#### Population

Studies that enrolled any registered healthcare providers were included. Subgroups of interest for our analyses included comparing physician participants to other healthcare providers (non-physicians). We excluded trials if less than 75 % of the participants included were certified, regulated healthcare providers.

#### Intervention

All studies that evaluated the impact of an intervention on the implementation of, uptake of, or adherence to a clinical practice guideline by a health care provider were included. The guideline of concern had to relate to the prevention or management of CVD, including risk factor management for any of: diabetes, dyslipidemia or hypertension. Guideline definitions were based on authors stating a guideline to be such. A study was deemed to be about the implementation or adherence to a guideline if the trial report explicitly stated that improving use of a clinical practice guideline was the focus of the intervention. Types of interventions included: academic detailing, audit and feedback, educational sessions, continuing medical education (CME) sessions, and ‘other’ (such as reminders or decision support systems).

#### Comparison group

We selected studies that included at least one control group. Comparison groups included usual care, a similar guideline implementation intervention of differing intensity or duration than the main intervention group, or no intervention (receipt of the intervention at a different time than the intervention group, such as after data collection).

#### Primary outcomes

We included trials that reported both a measure of guideline adherence and at least one clinical outcome. Measures of adherence included self-reported adherence, prescription review, and chart review. We included studies reporting any relevant clinical outcomes and considered the following groups of outcomes for analyses: mortality, hospitalizations, quality of life, and disease targets. Outcomes assessed at similar time points were combined in our analysis as short term (3–6 months), and long term (7 months or longer).

### Study selection and data extraction

Articles were screened based on title and abstract using the inclusion criteria, then based on full text by two independent reviewers. Discrepancies were resolved by consensus.

Data from included articles was extracted in duplicate by independent extractors. We extracted study characteristics (study design, setting and population), a description of the intervention (the type of intervention, providers, and resources involved), comparison intervention, risk of bias, outcome measurement and results, and funding for the study. Risk of bias was assessed using the Cochrane Risk of Bias tool for RCTs [10]. All discrepancies between extractors were resolved through consensus. Data was managed using spreadsheets created for each extractor. Authors were contacted after data extraction and consensus meetings were completed to request missing data and to check the accuracy of our extractions.

### Data analysis

We conducted descriptive analyses of included studies. We conducted meta-analyses (MA) for outcome results when there was sufficient clinical homogeneity across the studies. Clinical homogeneity was based on similar study characteristics (intervention type, outcome and follow-up point of interest). Meta-analyses were conducted in Review Manager (RevMan 5), using a random effects model and forest plots were generated. Intraclass correlation coefficient (ICC) for cluster RCTs were used in our meta-analyses to calculate the effective sample size to ensure the effect of clustering was taken into account in our analyses, as per the Cochrane Handbook [11]. A Z-test was used to assess statistical significance of meta-analysis results and a *p* < 0.05 was considered significant.

The adherence and patient outcomes were measured as both dichotomous and continuous outcome measures. Odds ratios (OR) and 95 % confidence intervals (CI) were calculated for use in the MA for dichotomous outcomes. Standardized mean differences (SMD) were used for continuous outcomes, as outcomes measuring the same construct were measured on different scales. Most continuous outcome analyses looked at the differences in mean change of each group from baseline, and this value was used in the MA, though some trials reported follow up results in each group, wherein we calculated the change score for each group to use in our MA. In order to impute the standard deviation for the change score in this instance, the standard deviation of the change score from another similar study was used.

We conducted sensitivity analyses to determine the robustness of our results, comparing the results of our analyses including and excluding studies with imputed standard deviations, and excluding studies with high risk of bias (greater than 3 domains rated as high risk of bias). We conducted subgroup analyses considering participant subgroups (physician participants and other healthcare providers), and considering the condition that was the focus of the guideline in the study (acute and chronic CVD conditions or risk factors). We planned to create funnel plots to investigate potential publication bias if at least ten studies were included for a given outcome, however this was not possible. We present a summary of the overall strength of evidence available using GRADE Summary of Findings tables produced using GRADEpro.

## Results

### Results of the search

**Fig. 1:**
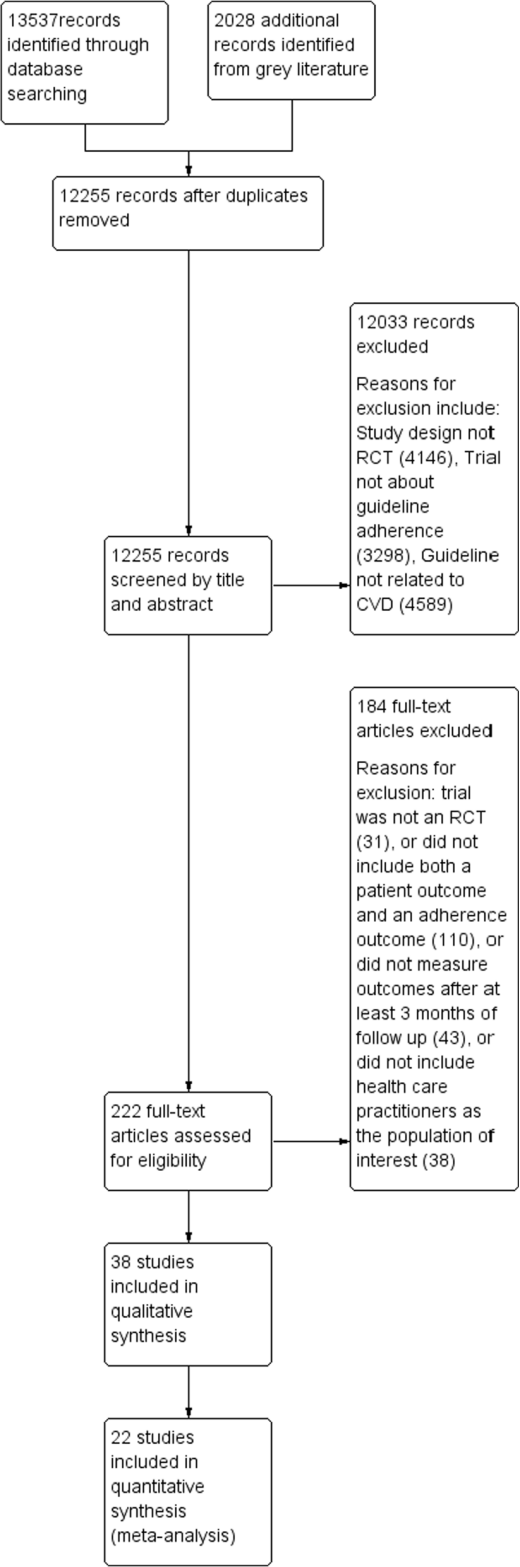
PRISMA flow chart of study inclusions

We identified 12,255 potentially relevant unique citations. We excluded 12,033 citations during the initial abstract and title screening. We reviewed 222 full text publications and included 38 studies in the review [12–54] (Fig. 1).

### Included studies (Table 1)

Eighteen studies took place in the USA, 14 were completed in Europe (the Netherlands, Italy, England, and Norway), two took place in Canada, one in South Africa, one in Brazil, one in Asia-Pacific area, and one in the Virgin Islands. Thirty-five studies included an intervention to improve physician use of guidelines and ten of those studies included a nurse as a target for the intervention; two studies focused on nurses alone, and one study focused on pharmacists. The most common intervention type was educational focused intervention (18/38), followed by audit and feedback (9/38), academic detailing focused interventions (4/38), comprehensive interventions that included education, audit and feedback and an academic detailing component (2/38), and “other” interventions that did not fall into any pre-designated category (8/38). Seven trials included more than one intervention group. All studies included an adherence outcome, as per our inclusion criterion. Disease target was the most common clinical outcome reported (33/38 trials), followed by mortality (11/38). Hospitalization and quality of life data were also reported in 8/38 and 6/38 trials, respectively.

### Risk of bias in included studies

**Fig. 2:**
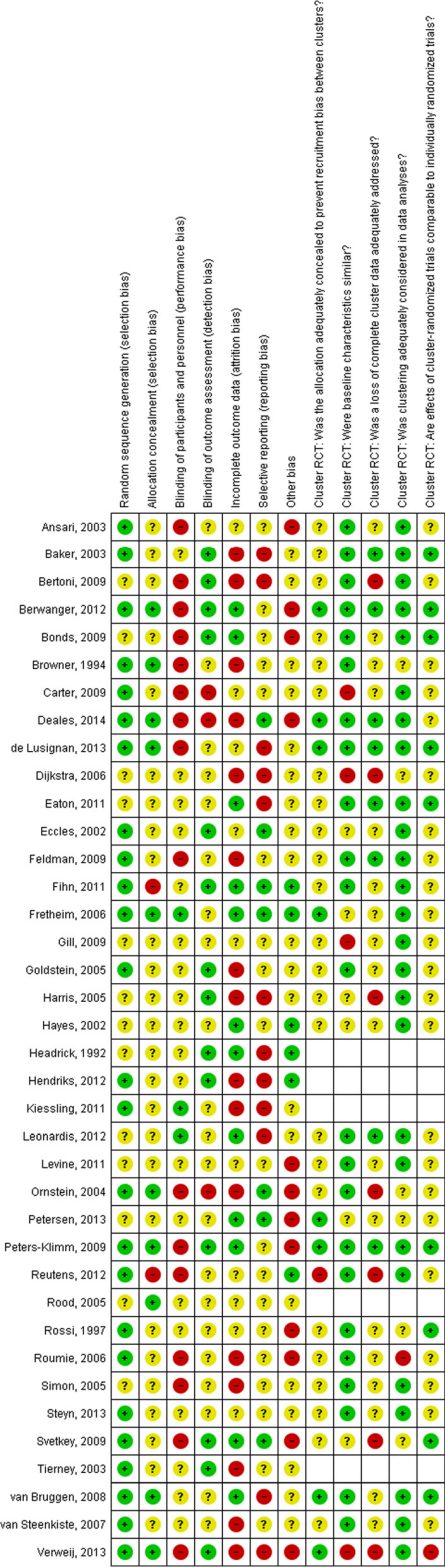
Risk of bias summary table for each study. Green indicates a low risk of bias, yellow indicates unclear risk of bias and red indicates high risk of bias, as assessed by reviewers using the Cochrane risk of bias tool

**Fig. 3:**
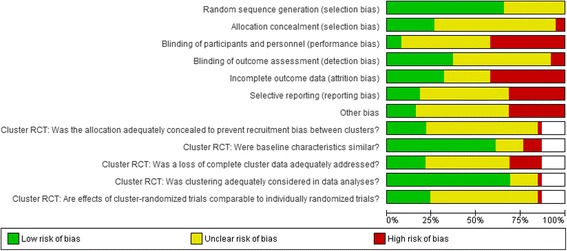
Risk of bias summary graph, summarized for each domain. Green indicates a low risk of bias, yellow indicates unclear risk of bias and red indicates high risk of bias, as assessed by reviewers using the Cochrane risk of bias tool

Risk of bias summary graphs and tables were created using RevMan (Figs. 2 and 3). Risk of bias was often assessed as unclear due to poor reporting of a methodological procedure. The majority of trials (33/38) were cluster RCTs, therefore additional risk of bias criteria were included for these studies. Random sequence generation was most often assessed to be low risk of bias, while blinding of participants was most commonly rated as high risk of bias.

### Effects of interventions

#### Education intervention

**Fig. 4:**
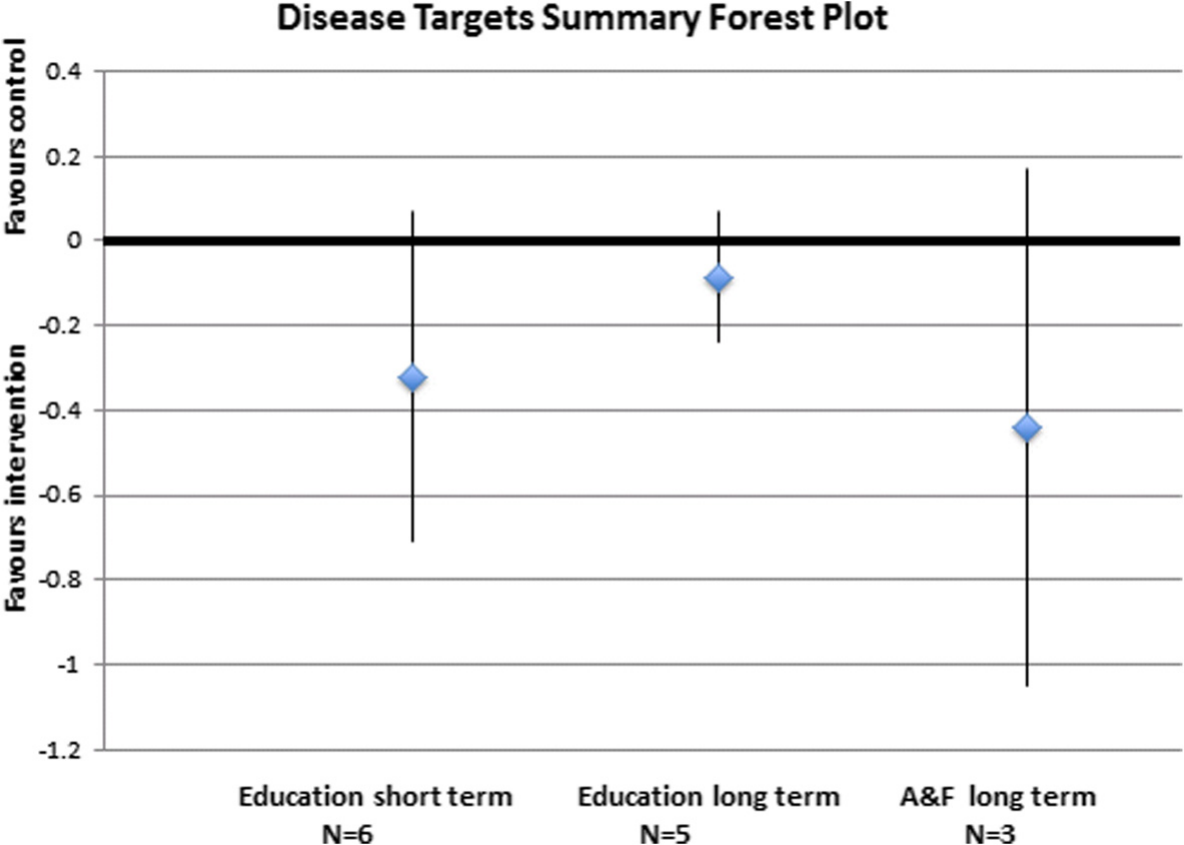
Forest plot of education intervention comparison for continuous adherence outcome at a short term time point (<6 months)

**Fig. 5:**
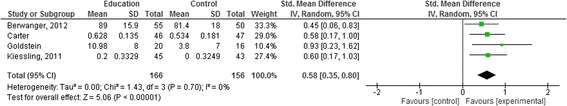
Summary disease target outcome forest plot for three comparisons measured by standardized mean difference, with point estimate and 95 % CIs

**Fig. 6:**
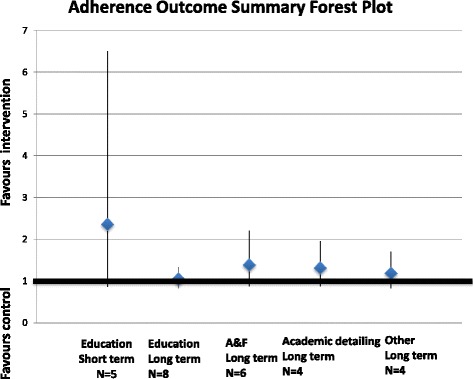
Summary adherence outcome forest plot for five comparisons measured by odds ratio, with point estimates and 95 % CIs

Seventeen trials tested an education-focused intervention and were included in a meta-analysis. These trials overall favoured the intervention, and one meta-analysis was statistically significant. Seven trials (2545 subjects) reported mortality outcomes, three of which reported mortality at a short term time point with an overall odds ratio of 0.54 (95 % CI 0.2 to 1.42). Four trials reported mortality at a long term time point with an overall odds ratio of 0.48 (95 % CI 0.11 to 1.98). Four trials (979 subjects) reported hospitalizations as an outcome at a long term time point. The overall odds ratio for this outcome was 0.88 (95 % CI 0.54 to 1.41). Six trials (2145 subjects) reported disease target results at a short term time point (SMD = −0.32 (95 % CI −0.71 to 0.07)) and five trials (2732 subjects) reported this outcome at a long term time point (SMD = −0.09 (95 % CI −0.24 to 0.07)) (Fig. 5). Seventeen trials reported adherence outcome data, six (2306 subjects) reported dichotomous data at a short term time point (OR = 2.11 (95 % CI 0.90 to 4.97)), four trials (322 subjects) reported continuous data at a short term time point (Fig. 4) and eight trials (6019 subjects) reported dichotomous data at a long term time point (OR = 1.05 (95 % CI 0.82 to 1.34)) (Fig. 6).

#### Audit and feedback

Nine trials included an intervention that focused on audit and feedback, and seven of those trials reported data sufficient to be included in meta-analyses. Three trials (2240 subjects) reported disease target results at a long term time point with an overall effect of −0.44 SMD (95 % CI −1.05 to 0.17) (Fig. 5). Six trials (2983 subjects) reported adherence data at a long term time point with an overall odds ratio of 1.39 (95 % CI 0.88 to 2.21) (Fig. 6).

#### Academic detailing

Four trials (6017 subjects) included academic detailing as the focus of the intervention and all of these trials reported data that was included in a meta-analysis for adherence outcome. The overall odds ratio for this comparison was 1.32 (95 % CI 0.88 to 1.96) (Fig. 6).

#### Other interventions

Eight trials included an intervention whose focus did not fit these previous groups. Four trials (1782 subjects) included a decision support tool as the focus of their intervention. The overall odds ratio for this comparison was 1.19 (95 % CI 0.83 to 1.70) (Fig. 6).

### Sensitivity and subgroup analyses

Sensitivity analysis investigating the impact of imputed standard deviations in continuous data was possible in the education intervention outcome for the disease target outcome at a short term time point. The pooled SMD from six studies in this comparison was −0.32 (95 % CI −0.71 to 0.07), while the estimate from the sensitivity analysis, with studies that included imputed standard deviation removed, was −0.27 (95 % CI −0.71 to 0.17). Another sensitivity analysis, investigating the impact of high risk of bias studies on the overall estimate was possible for the meta-analysis of the effect of education on short term adherence outcomes. The pooled odds ratio was 2.36 (95 % CI 0.86 to 6.51) before studies with high risk of bias were excluded, and 3.65 (95 % CI 0.53 to 25.15) after studies with high risk of bias were excluded.

We compared results in studies that targeted physicians only in their intervention to interventions that involved non-physician healthcare providers alone or in addition to physicians with subgroup analysis. This subgroup analysis was possible in seven comparisons, and the subgroups of physician participants alone frequently had less heterogeneity than when grouped with all studies, suggesting participants may be a source of heterogeneity (Additional file 2: Figure S1). Another subgroup analysis we conducted compared results in studies that focused on an acute cardiovascular condition to a chronic cardiovascular condition. Five comparisons showed inconsistent results although the heterogeneity was reduced in at least one of the two subgroups in all comparisons.

### GRADE summary of findings tables

The overall quality of evidence identified in this systematic review was moderate to very low due to high risk of bias, imprecision, and heterogeneity (Table 2). The most patient important outcome of mortality had moderate quality of evidence associated, indicating the results may be interpreted with some confidence.

**Table 1:** Characteristics of included studies

Study ID	Topic of trial	Study Design	Population description	Setting	Intervention Description; Intervention 2 description (if applicable)	Type	Duration of treatment period	Comparison intervention	Outcomes measured	Risk of bias rating^a^
Ansari, 2003	Use of beta-blockers in congestive heart failure	cRCT	Specialist doctors and nurse practitioners, patients with CHF	USA, urban medical centre	Nurse facilitator plus healthcare provider educational sessions; provider and patient reminder letters	Other type: Nurse facilitator; notifications	1 year	Educational sessions, no nurse facilitator	Mortality, hospitalization, adherence (prescription review, chart review)	High risk of bias
Baker, 2003	Guidelines in prioritised review criteria	cRCT	Family doctors, patients with angina	England, general practices	Review criteria; criteria plus feedback	Other type: review criteria	12 months	Guideline dissemination alone	Disease target (cholesterol), adherence (prescription review, chart review)	Low risk of bias
Bertoni, 2009	Physician adherence to ATP III guidelines	cRCT	Family doctors	USA, primary care practices	CDSS, educational sessions, academic detailing, CME sessions	Education + audit and feedback + academic detailing + CME session	2 years	educational sessions, CME sessions, guideline mailed to participants	Disease target (cholesterol), adherence (prescription review, chart review)	High risk of bias
Berwanger, 2012	Multifaceted quality improvement intervention in ACS patients	cRCT	Patients with ACS at general public hospitals	Brazil, public hospitals	Training, reminders, checklists, case management, educational sessions	Education	8 months	Routine care	Mortality, major adverse cardiac events, adherence (prescription review)	Low risk of bias
Bonds, 2009	Compliance to JNC 7 guidelines to improve blood pressure	cRCT	Family doctors	USA, primary care practices	Educational sessions, dissemination of guidelines, academic detailing for physicians, feedback on blood pressure control	Education + audit and feedback + academic detailing + CME sessions	2 years	Similar to intervention but focused on ATPIII guidelines	Disease target (BP), adherence (prescription review, chart review)	Low risk of bias
Browner, 1994	CME and follow up to improve detection and treatment of high cholesterol	cRCT	Family and internal medicine doctors	USA, general practices	CME seminar; Intensive CME (office visits and educational materials)	Education + CME sessions	18 months	Educational sessions	Disease target (cholesterol), adherence (chart review)	High risk of bias
Carter, 2009	Physician and pharmacist collaborative model to improve blood pressure	cRCT	Family doctors, patients with hypertension	USA, community based family medicine	Collaborative model, team building exercises, training sessions, educational sessions	Education + other (collaborative model)	6 months	Collaborative model	Disease target (BP), guideline adherence tool	High risk of bias
De Lusignan, 2013	Audit based education to reduce blood pressure	cRCT	Mixed health care professionals	United Kingdom, primary care	Audit based education consisting of workshops; academic detailing plus workshops	Education + audit and feedback; academic detailing	2 years	Usual care	Mortality, major adverse cardiac events, disease target (BP), adherence (prescription review)	Low risk of bias
Deales, 2014	Team based approach to disease and care management	cRCT	Mixed health care professionals	Italy, primary care groups	Recommendations as textbooks and decision algorithms, education sessions	Education	12 months	Usual care	Disease target (HbA1c), adherence (chart review)	High risk of bias
Dijkstra, 2006	Implementation strategies for diabetes guidelines	cRCT	T1D and T2D patients	The Netherlands, hospitals	Educational meetings, feedback, reminder card; diabetes passport, education	Education + audit and feedback	1 year	Usual care	Disease target (HbA1c), adherence (chart review)	High risk of bias
Eaton, 2011	Multimodal intervention to improve screening and management of hyperlipidemic patients	cRCT	Family doctors	USA, primary care practices	PDA with decision support and education toolkit and academic detailing	Academic detailing	12 months	PDA with decision support but minimal follow up	Disease target (cholesterol), adherence (chart review)	Low risk of bias
Eccles, 2002	Computerised decision support system to implement angina guidelines	cRCT	Family doctors	England, general practices	Computer decision support that provided access to guidelines	Other: CDSS	12 months	Same intervention but asthma guideline provided	Quality of life, adherence (chart review)	Low risk of bias
Feldman, 2009	Simplified algorithm for treatment of hypertension	cRCT	Family practices, patients with hypertension	Canada, family practices	Algorithm, aids, one follow up meeting, educational materials and sessions	Education + Other (algorithms)	6 months	Educational sessions and guidelines	Mortality, disease target (BP), adherence (chart review)	Low risk of bias
Fihn, 2011	Collaborative care model based intervention to improve angina management	cRCT	Family doctors, patients with angina	USA, academic primary care clinics	Expert advice, progress evaluations, education	Education	12 months	Usual care	Mortality, disease target, adherence (chart review)	Low risk of bias
Fretheim, 2006	Tailored intervention to support implementation of CVD guidelines	cRCT	Family practices, hypertensive or hypercholesterolemic patients	Norway, general practices	Tailored intervention including reminders, audit and feedback and education	Education + audit and feedback	12 months	Passive dissemination	Disease target (cholesterol, BP), adherence (prescription review, chart review)	Low risk of bias
Gill, 2009	EMR-based intervention for lipid management	cRCT	Family doctors, general internists	USA, academic family practice	EMR disease management tool	Other (integration into EMR)	12 months	Usual care	Disease target (cholesterol), adherence (chart review)	High risk of bias
Goldstein, 2005	Intervention on drug choice for hypertension	cRCT	Family doctors, nurse practitioners	USA, multiple sites	Education, individual drug profiles, follow up	Education	9 months	Education on guidelines	Disease target (BP), adherence (prescription and chart review)	Low risk of bias
Harris, 2005	Teleconferenced educational detailing for diabetes	cRCT	Family doctors	Canada, family practices	Eight one hour small group educational sessions with opinion leaders	Education	3 months	CME session after intervention period	Disease target (HbA1c), adherence (chart review)	High risk of bias
Hayes, 2002	Quality improvement and written feedback for CHF management	cRCT	Hospitals, CHF patients	USA, hospitals	Education, quality improvement tools from liaisons, chart reminders	Education + audit and feedback	6 months	Mailed quality improvement tools	Disease target (ventricular fxn), adherence (chart review)	High risk of bias
Headrick, 1992	Education and feedback strategies to improve compliance with NCEP-PCEP guidelines	RCT	Resident doctors	USA. Academic hospital	Lecture, chart reminders; Lecture, patient specific feedback and chart reminder	Education + Other (reminders)	20 weeks	Lecture alone	Disease targets (cholesterol), adherence (chart review)	Low risk of bias
Hendriks, 2012	Nurse led guideline based software supported ICCP	RCT	Family doctors, specialists, patients with atrial fibrillation	Netherlands, academic center	Nurse specialist educated patients and CDSS	Other (nurse specialist)	12 months	Usual care	Mortality, hospitalizations, quality of life, adherence (chart review)	Low risk of bias
Kiessling, 2011	Case based training to optimize hyperlipidemia care	RCT	Family doctors, patients with CHD	Sweden, primary health care centres	Case based training seminars and guideline provided	Education	2 years	Usual care	Mortality, disease target (cholesterol), adherence (prescription review)	High risk of bias
Leonardis, 2012	Multimodal intervention to improve adherence to targets	cRCT	Specialists, CKD patients	Italy, renal clinics	Education session, follow up and audits	Education + audit and feedback	3 years	Education and standard care	Mortality, hospitalizations, quality of life, disease target (cholesterol), adherence (prescription/ chart review)	Low risk of bias
Levine, 2011	Multicomponent internet delivered intervention improve CHD guideline adherence	cRCT	Family doctors, MI patients	Virgin Islands and Puerto Rico, community primary care clinics	Educational cases, guidelines, monthly update, reminders	Education + Other (reminders)	27 months	Passive dissemination	Disease target (cholesterol), adherence (chart review)	High risk of bias
Ornstein, 2004	Multimethod quality improvement intervention for adherence to quality indicators in CVD and stroke	cRCT	Practice based research network of practices	USA, primary care practices	Education, performance reports quarterly, practice site visits and network meetings (6–7 1–2 day visits) with pharmacist (academic detailing)	Education + academic detailing	2 years	Education, performance reports quarterly	Disease target (BP), adherence (prescription, chart review)	High risk of bias
Petersen, 2013	Effect of financial incentives to reward guideline based hypertension care	cRCT	Family doctors	USA, primary care clinics	Physician level incentives; practice levels incetives; combined (both) incentives	Other (incentives)	20 months	Usual care	Disease target (BP), adherence (prescription, chart review)	High risk of bias
Peters-Klimm, 2009	Educational model for GPs for the management of CHF	cRCT	Family doctors, CHF patients	Germany, general practitioner clinics	“Train the trainer” = multidisciplinary andragogic and didactic educational sessions	Education + Other (feedback)	7 months	Single educational session by cardiologist	Mortality, hospitalizations, quality of life, disease target (course), adherence (prescription review)	Low risk of bias
Reutens, 2012	Education of GPs on the IDF-WPR guidelines to improve metabolic control	cRCT	Family doctors, T2D patients	Asia-Pacfic, general practitioner clinics	Education meetings (two 3 months apart), reminder letters and cards, flowsheet on patient notes, patient diabetes passport	Education + Other (reminders, diabetes passport)	12 months	Instructed on assessments in study but no information on guidelines	Disease target (BP), adherence (chart review)	High risk of bias
Rood, 2005	Computer based guidelines to improve nurse measurement of patient glucose	RCT	ICU patients	The Netherlands, teaching hospital	Guideline based advice via computer decision support software	Other (decision support tool)	10 weeks	Paper based guideline flowchart	Disease target (glucose), adherence (chart review)	High risk of bias
Rossi, 1997	Guideline reminders to improve prescribing based on JNC V guideline	cRCT	Nurse practitioners, hypertension patients	USA, GIM clinic	Guideline reminder for prescription and alternatives	Other (reminder)	5 months	Usual care	Disease target (BP), adherence (prescription review)	High risk of bias
Roumie, 2006	Multifactorial intervention to improve quality of care of hypertension patients	cRCT	Physicians and nurse practitioners, hypertension patients	USA, community and hospital clinics	Alert on medical record; Educational sessions and alert on medical record	Education + other (alerts)	6 months	Providers received email with guideline	Mortality, hospitalizations, disease target (BP), adherence (prescription review)	High risk of bias
Simon, 2005	Academic detailing individually or group to increase diuretic use in hypertension patients	cRCT	Family doctors, hypertension patients	USA, community health plan	Academic detailing meeting one-on-one; small group academic detailing session	Academic detailing	3 months	Passive dissemination	Hospitalizations, disease target(BP), adherence (chart review)	High risk of bias
Steyn, 2013	Structured clinical record and training health care providers to control diabetes and hypertension	cRCT	Nurses, patients with diabetes and hypertension	South Africa, community health centres	Structured record with guideline embedded added to patient folders, educational package	Education	1 year	Passive dissemination	Disease target (HbA1c), adherence (chart review)	High risk of bias
Svetkey, 2009	Intervention to increase physician adherence to BP guideline	cRCT	Physicians, hypertension patients	USA, community practice	CME courses, treatment algorithm, quarterly feedback on adherence	Education + CME session + other (feedback)	18 months	Usual care	Disease target (BP), adherence (chart review)	Low risk of bias
Tierney, 2003	Decision support system with guideline for managing ischemic heart disease and CHF patients	RCT	Pharmacists, CHF patients	USA, academic primary care practice	Physicians received patient specific feedback; pharmacist system to send feedback to physicians; both	Education + audit and feedback + other (decision support system)	1 year	Usual care	Mortality, hospitalizations, quality of life, adherence (chart review)	High risk of bias
Van Bruggen, 2008	Facilitator enhanced multifaceted intervention for T2D guideline implementation	cRCT	Family doctors and nurses and practice assistants, T2D patients	The Netherlands, primary care practices	Facilitators visited twice a month to train staff on guidelines, performance feedback,	Education + audit and feedback	1 year	Usual care	Disease target (HbA1c), adherence (prescription and chart review)	Low risk of bias
Van Steenkiste, 2007	Decision support tool for risk management improving CVD guideline performance	cRCT	Family doctors, patients without CVD	The Netherlands, hospital	Education, decision support tool,	Other (decision support tool)	8 months	Educational materials on guideline	Disease target (lifestyle), adherence (chart review)	High risk of bias
Verweij, 2013	Effectiveness of guideline based care on weight, CVD risk	cRCT	Occupational physicians	The Netherlands, occupational medicine	Environment scan, patient counselling training, patient toolkit	Other (environment scan, toolkit)	18 months	Usual care	Quality of life, disease target (BP), adherence (chart review)	High risk of bias

Footnote: ^a^ Risk of bias rated as high or low risk of bias based on overall domains, where high risk of bias designated if greater than 3 domains rated as high risk of bias

**Table 2:** Summary of findings table for educational interventions

Education compared to control for improving adherence to cardiovascular disease guidelines						
Patient or population: patients with improving adherence to cardiovascular disease guidelines						
Settings:						
Intervention: Education						
Comparison: control						
Outcomes	Illustrative comparative risks^a^ (95 % CI)		Relative effect	No of participants	Quality of the evidence	Comments
	Assumed risk control	Corresponding risk Education	(95 % CI)	(studies)	(GRADE)	
Mortality	Study population		OR 0.54	2190	⊕ ⊕ ⊕⊝	
Follow-up: median 6 months	40 per 1000	22 per 1000	(0.2 to 1.42)	(3 studies)	moderate^c^	
		(8 to 56)				
	Moderate					
	26 per 1000	14 per 1000				
		(5 to 37)^b^				
Disease Targets		The mean disease targets in the intervention groups was0.32 standard deviations lower		2145	⊕⊝⊝⊝	SMD −0.32 (−0.71 to 0.07)
Follow-up: 3–6 months				(6 studies)	very low^c,e,f^	
		(0.71 lower to 0.07 higher)				
Adherence		The mean adherence in the intervention groups was 0.58 standard deviations higher		322	⊕ ⊕ ⊕⊕	SMD 0.58 (0.35 to 0.8)
Follow-up: 6–24 months				(4 studies)	high	
		(0.35 to 0.8 higher)				
Mortality	Study population		OR 0.48	355	⊕ ⊕ ⊝⊝	
Follow-up: 7 months - 10 years	182 per 1000	96 per 1000	(0.11 to 1.98)	(4 studies)	low^g^	
		(24 to 306)				
	Moderate					
	146 per 1000	76 per 1000				
		(18 to 253)^b^				
Hospitalizations	Study population		OR 0.88	979	⊕ ⊕ ⊕⊕	
Follow-up: 7–22 months	188 per 1000	170 per 1000	(0.54 to 1.41)	(4 studies)	high	
		(111 to 246)				
	Moderate					
	191 per 1000	172 per 1000				
		(113 to 250)^b^				
Disease Targets		The mean disease targets in the intervention groups was 0.09 standard deviations lower		2732	⊕ ⊕ ⊝⊝	SMD −0.09 (−0.24 to 0.07)
Follow-up: 7–27 months				(5 studies)	low^f,h^	
		(0.24 lower to 0.07 higher)				
Adherence	Study population		OR 1.05	6019	⊕ ⊕ ⊝⊝	
Follow-up: 7–27 months	609 per 1000	620 per 1000	(0.82 to 1.34)	(8 studies)	low^c,i^	
		(561 to 676)				
	Moderate					
	236 per 1000	245 per 1000				
		(202 to 293)^b^				
Adherence	Study population		OR 2.36	2145	⊕⊝⊝⊝	
Follow-up: median 6 months	288 per 1000	489 per 1000	(0.86 to 6.51)	(5 studies)	very low^c,j,k^	
		(258 to 725)				
	Moderate					
	326 per 1000	533 per 1000				
		(294 to 759)^b^				

^a^The basis for the assumed risk (e.g. the median control group risk across studies) is provided in footnotes. The corresponding risk (and its 95 % confidence interval) is based on the assumed risk in the comparison group and the relative effect of the intervention (and its 95 % CI)

*CI* Confidence interval, *OR* Odds ratio

GRADE Working Group grades of evidence

High quality: Further research is very unlikely to change our confidence in the estimate of effect

Moderate quality: Further research is likely to have an important impact on our confidence in the estimate of effect and may change the estimate

Low quality: Further research is very likely to have an important impact on our confidence in the estimate of effect and is likely to change the estimate

Very low quality: We are very uncertain about the estimate

^b^Assumed Risk is based on the default calculation within GRADEpro (mean control group risk, and median control group risk)

^c^Assessment based on three studies thus precision cannot be accurately determined

^d^Several included studies had 3 or more high risk of bias assessments

^e^Statistical heterogeneity I2 = 94 %

^f^Disease targets are an indirect estimate of patient important outcomes

^g^Statistical heterogeneity I2 = 70 %

^h^Statistical heterogeneity I2 = 41 %

^i^Statistical heterogeneity I2 = 60 %

^j^Statistical heterogeneity I2 = 95 %

^k^Overall estimate has large range for 95 % confidence interval

## Discussion

### Statement of principal findings

We have focused on interventions aimed at improving adherence to CVD guidelines. Overall studies are variable in their conclusions on whether the intervention was effective, though our quantitative analysis supports that interventions trend towards having an impact on adherence to guidelines and patient outcomes. One comparison of an education intervention for the adherence outcome was statistically significant, indicating this area of study deserves further consideration, as these interventions may help improve both adherence to guidelines, and more importantly, patient outcomes. Our results were robust where sensitivity analyses were possible. Subgroup analyses (participant and condition) reduced the statistical heterogeneity but there was inconsistency in the subgroup with the larger effect for each analysis. In some cases, the physician subgroup favoured the intervention to a greater degree than the non-physician subgroup, but in other comparisons the opposite was true. The same results were found for the condition subgroup (acute vs. chronic condition). The confidence in these recommendations ranged from moderate to very low based on a GRADE summary of findings due to imprecision, risk of bias, and inconsistency.

### Strengths and weaknesses of the review

Our systematic review has several strengths, including that it was comprehensive in inclusion of studies. We included all types of healthcare providers in order to illustrate the impact these interventions can have on both physicians and non-physicians, which is increasingly important for multidisciplinary teams required for complex diseases such as CVD. We limited our study inclusion to those that reported both adherence and a patient outcome, as interventions must improve both in order to be clinically useful. All screening, data extraction, and risk of bias assessment was done in duplicate with trained reviewers to ensure the reproducibility of these results. Our quantitative analysis was pre-specified to avoid finding spurious results due to post hoc analyses. We minimized the number of comparisons that were made while ensuring comparisons had fairly good clinical homogeneity to maintain the strength of those conclusions. We also contacted authors for missing data and to verify the accuracy of our data extractions of their trial, thus we have confidence in this data.

However, this review has limitations. The first relates to the quality of reporting in trials. Reporting of risk of bias domains was poor in many trials, making it difficult to assess risk of bias. There was also significant heterogeneity in the studies’ interventions and characteristics making combining results in a meta-analysis difficult, leading to small numbers of studies included in each comparison. Meta-analyzing results was further complicated by uncertainty of the exact nature of some interventions due to limited descriptions of interventions available in publications. This also limited our ability to assess publication bias, so we were unable to determine the effect that might have on our confidence in these results.

### Comparison to similar reviews

A systematic review on CVD guideline implementation strategies in primary care physicians by Unverzagt et al. reported similar conclusions on the effectiveness of education and reminder system interventions to improve adherence [9]. Our review extends these findings, illustrating the impact at the patient level on mortality, hospitalizations, quality of life and disease targets, and to different healthcare providers.

Similar to our findings, a review by Grimshaw et al. on guideline implementation noted overall the most effective interventions tend to include specific educational interventions and patient specific reminders at point of care [6].

### Meaning of the review results

These results indicate there is some evidence to support the use of some interventions to improve healthcare provider adherence to CVD guidelines. Despite the limitations in the studies in this review, a trend of interventions improving adherence and patient outcomes was noted, supporting that these interventions may be more effective than passive guideline dissemination strategies. However, more studies are needed to strengthen these conclusions.

The majority of interventions included were multifaceted, which some reviews have suggested provide positive outcomes more frequently than single interventions [53–55]. However, our results were not consistent with these; we found these interventions have limited effects, which may be related to the number of components in a given intervention, as only two interventions included all of the types of interventions. A review by Squires et al. found there is ambiguity in the evidence of whether multifaceted interventions are more effective than single interventions, which is in agreement with our inconsistent findings [55].

Another possible reason for the overall small effect sizes may relate to the complexity of the management of CVD. This includes treating and preventing multiple risk factors in patients, such as diabetes, hypertension and dyslipidemia [56]. Most guidelines address only one of these diseases, and this may contribute to the small improvements found in this review. Given the multifactorial nature of CVD, it needs to be treated with guidelines that acknowledge this. Using harmonized CVD guidelines such as C-CHANGE is an important step that needs to be taken in CVD guideline implementation intervention trials to ensure the best, most comprehensive care is provided to patients [1]. This is also an important consideration as to why CVD guideline implementation strategies must differ from strategies used in treating simpler diseases such as pneumonia or asthma [57, 58].

### Unanswered questions and future research

It would be beneficial for more high quality studies on this topic to be conducted to improve the strength of our recommendations, given the low confidence in most of these estimates due to a small number of studies included in each MA. Interventions should be fully described so they are not only reproducible, but future reviews are able to confidently determine homogeneous groups for meta-analyses. Future reviews on this topic should also define clinically important differences to determine whether the effects are not only statistically significant, but clinically significant as well.

## Conclusions

Interventions to improve adherence to CVD guidelines can be effective at improving both adherence and patient outcomes, and are often more effective than guideline dissemination alone. Interventions that focused on healthcare provider education demonstrated statistically significant improvements. The overall quality of evidence available in this review was low, but several patient important outcomes including mortality were supported by moderate to high quality evidence.

## Additional files

Additional file 1:
**Supplementary: Medline search strategy.** (DOCX 14 kb) Additional file 2: Figure S1. Figure A: Subgroup analysis of physician participants compared to other participants in education focused intervention trials disease target outcome at a short term time point. (DOCX 30 kb)

## Abbreviations

CVD: Cardiovascular disease
CPG: Clinical practice guideline
MA: Meta-analysis
RCT: Randomized controlled trials
GRADE: Grading of Recommendations Assessment, Development and Evaluation
CME: Continuing medical education
OR: Odds ratio
SMD: Standardized mean difference
CI: Confidence interval
