# Supplementary material for: Interventions to improve adherence to cardiovascular disease guidelines: a systematic review
Source: BMC Fam Pract. 2015 Oct 22;16:147. doi: 10.1186/s12875-015-0341-7 (PMC4619086; doi:10.1186/s12875-015-0341-7)
Supplement: Additional file 1: — Supplementary: Medline search strategy. (DOCX 14 kb) [file 12875_2015_341_MOESM1_ESM.docx]

**Additional file 1: Supplementary online: Medline Search strategy**

1. Guideline Adherence [Mesh]
2. Adheren*[tw]
3. Uptake[tw]
4. Implement*[tw]
5. Adopt*[tw]
6. “Diffusion of Innovation”[Mesh:NoExp]
7. 1 OR 2 OR 3 OR 4 OR 5 OR 6
8. “Practice Guidelines as Topic”[Mesh]
9. “practice guideline*”[Publication Type] OR “practice guideline*”[AllFields]
10. "practice guideline"[Publication Type] OR "practice guidelines as topic"[MeSH Terms] OR "clinical practice guideline"[All Fields] OR "Practice Guidelines as Topic"[Mesh]
11. “cardiovascular diseases”[Mesh Terms]
12. Cardiovascular diseases[tw]
13. Cardio*[tw]
14. Cardia*[tw]
15. Heart*[tw]
16. Coronary*[tw]
17. Angina*[tw]
18. Ventric*[tw]
19. Myocard*[tw]
20. Isch?em*[tw]
21. Emboli*[tw]
22. Thombo*[tw]
23. Endocardi*[tw]
24. Stroke[Mesh]
25. Stroke*[tw]
26. Cerbrovasc*[tw]
27. Cerebral vascular[tw]
28. Brain accident**[tw]
29. Brain infarct*[tw]
30. Cerebral infarct*[tw]
31. Hypertension[Mesh]
32. Hypertensi*[tw]
33. Peripheral arter* disease[tw]
34. “blood pressure, high”[tw]
35. Elevated blood pressure [tw]
36. Dyslipidemias[Mesh]
37. Hyperlipid*[tw]
38. Hypercholesterol*[tw]
39. Hyperlipidemia*[tw]
40. Hypercholesteremia*[tw]
41. Hypertriglyceri*[tw]
42. Hyperlipoproteinemia*[tw]
43. Diabetes mellitus [Mesh]
44. Diabet*[tw]
45. Arteriosclerosis[Mesh]
46. Cholesterol[Mesh]
47. Coronary risk factor*[tw]
48. Blood pressure[Mesh]
49. Blood pressure[tw]
50. 11 OR 12…. OR 49
51. Randomized controlled trial[pt]
52. Controlled clinical trial[pt]
53. Randomized[tiab]
54. Placebo[tiab]
55. Drug therapy[sh]
56. Randomly[tiab]
57. Trial[tiab]
58. Groups[tiab]
59. 51 OR 52 OR 53 OR 54 OR 55 OR 56 OR 57 OR 58
60. Animals[mh] NOT humans[mh]
61. 7 AND 10 AND 50 AND 59 NOT 60
