# Supplementary material for: Interventions to improve adherence to cardiovascular disease guidelines: a systematic review
Source: BMC Fam Pract. 2015 Oct 22;16:147. doi: 10.1186/s12875-015-0341-7 (PMC4619086; doi:10.1186/s12875-015-0341-7)
Supplement: Additional file 2: Figure S1. — Figure A: Subgroup analysis of physician participants compared to other participants in education focused intervention trials disease target outcome at a short term time point. (DOCX 30 kb) [file 12875_2015_341_MOESM2_ESM.docx]

**Additional file 2: Supplementary online figures:**


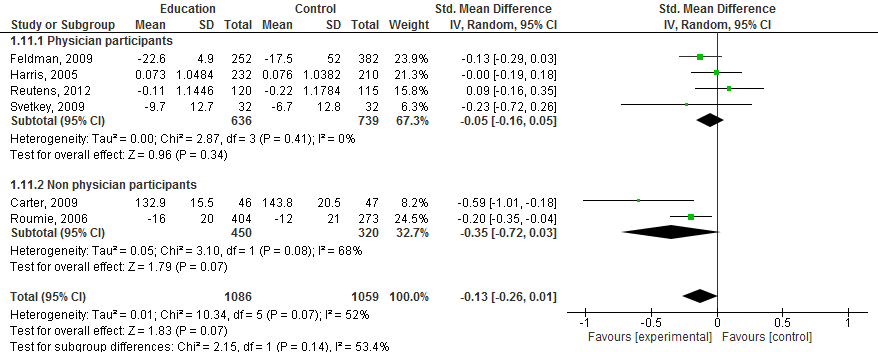


Figure A: Subgroup analysis of physician participants compared to other participants in education focused intervention trials disease target outcome at a short term time point
